# Inflammatory Regulation by TNF-α-Activated Adipose-Derived Stem Cells in the Human Bladder Cancer Microenvironment

**DOI:** 10.3390/ijms22083987

**Published:** 2021-04-13

**Authors:** Hui-Kung Ting, Chin-Li Chen, En Meng, Juin-Hong Cherng, Shu-Jen Chang, Chien-Chang Kao, Ming-Hsin Yang, Fang-Shiuan Leung, Sheng-Tang Wu

**Affiliations:** 1 Division of Urology, Department of Surgery, Tri-Service General Hospital, National Defense Medical Center, Taipei 114, Taiwan; giu0114@gmail.com (H.-K.T.); j0921713355@gmail.com (C.-L.C.); en.meng@gmail.com (E.M.); guman2011@gmail.com (C.-C.K.); yangming@mail.ndmctsgh.edu.tw (M.-H.Y.); 2 Department and Graduate Institute of Biochemistry, National Defense Medical Center, Taipei 114, Taiwan; 3 Department and Graduate Institute of Biology and Anatomy, National Defense Medical Center, Taipei 114, Taiwan; i72bbb@gmail.com; 4 Department of Gerontological Health Care, National Taipei University of Nursing and Health Sciences, Taipei 112, Taiwan; 5 Laboratory of Adult Stem Cell and Tissue Regeneration, National Defense Medical Center, Taipei 114, Taiwan; belle661011@gmail.com; 6 College of Biological Science, University of California-Davis, Davis, CA 95616, USA; sherry31748@gmail.com

**Keywords:** adipose-derived stem cells, immunomodulatory, tumor necrosis factor-α, tumorigenicity, bladder cancer, inflammatory microenvironment

## Abstract

Mesenchymal stem cells (MSCs), such as adipose-derived stem cells (ADSCs), have the most impressive ability to reduce inflammation through paracrine growth factors and cytokines that participate in inflammation. Tumor necrosis factor (TNF)-α bioactivity is a prerequisite in several inflammatory and autoimmune disease models. This study investigated the effects of TNF-α stimulate on ADSCs in the tumor microenvironment. The RNAseq analysis and cytokines assay demonstrated that TNF-α stimulated ADSCs proliferation and pro-inflammatory genes that correlated to leukocytes differentiation were upregulated. We found that upregulation of TLR2 or PTGS2 toward to IRF7 gene-associated with immunomodulatory and antitumor pathway under TNF-α treatment. In TNF-α-treated ADSCs cultured with the bladder cancer (BC) cell medium, the results showed that apoptosis ratio and OCT-4 and TLR2 genes which maintained the self-renewal ability of stem cells were decreased. Furthermore, the cell survival regulation genes including TRAF1, NF-kB, and IRF7 were upregulated in TNF-α-treated ADSCs. Additionally, these genes have not been upregulated in BC cell medium. A parallel study showed that tumor progressing genes were downregulated in TNF-α-treated ADSCs. Hence, the study suggests that TNF-α enhances the immunomodulatory potential of ADSCs during tumorigenesis and provides insight into highly efficacious MSC-based therapeutic options for BC.

## 1. Introduction

Bladder cancer (BC) is the ninth predominant cancer among the most common cancers worldwide, characterized by high recurrence of aggressive disease [1]. In addition to several causes, chronic inflammation is recognized as a risk factor for BC [2]. Many types of cells are involved during a complex inflammation phase, including mesenchymal stem cells (MSCs). MSCs play vital roles as they affect the inflammatory microenvironment by modulating the immune response and secretion of growth factors and cytokines, as well as the release of a wide range of immunoregulatory factors [3,4,5].

MSCs are multipotent cells derived from embryonic connective tissue (mesenchyme) that is derived from the mesoderm; they can therefore differentiate into a variety of cells from the mesoderm, such as chondrocytes, osteocytes, adipocytes, smooth muscle cells, and some other types of germ layer cells, including neurons and hepatocytes [6,7,8,9,10]. In recent years, MSCs have been acknowledged as adult stem cells that actively participate in regeneration processes in damaged tissue [11]. It has been suggested that they originate from the bone marrow or located in the surroundings which begin to immediately migrate to the injury site and extensively cooperate with various types of stromal and inflammatory cells [12]. MSCs can regulate a variety of soluble factors and exert immunomodulatory, angiogenic, antiapoptotic, and antioxidative effects via cell–cell interaction and paracrine activities [13]. Thus, MSCs play a crucial role in the inflammatory cascade and have recently emerged as key components of cell therapy targeted toward autoimmune, inflammatory, and degenerative diseases.

MSCs may exert distinct immunomodulatory effects depending on the regulatory cytokines or growth factors released during their interaction with immune cells [14,15]. They actively sense the inflammation or tumor microenvironment and modulate the function of the host’s immune cells, depending on the local immune cells [4]. Inflammatory stimulation provides MSCs with the ability to suppress or boost the immune response, depending on the molecular mechanisms involved, thereby enhancing the plasticity of the immunomodulatory effect of MSCs [8]. For example, the Toll-like receptors (TLRs) associated with nuclear factor (NF)-κB signaling differentially modulate the proliferation and survival of MSCs [16] during inflammation [17]. Further, a vital component in the immune system during inflammation is the inflammatory response in which tumor necrosis factor-α (TNF-α) involved. TNF-α, which is expressed in ischemic and injured tissues, contributes not only in inflammatory response but also in repair processes [18,19]. Previous studies have shown that TNF-α-treated human adipose tissue-derived MSCs secreted various protein factors, such as cytokines, extracellular matrix, proteases, and protease inhibitors as well as hastened cutaneous wound healing and angiogenesis through IL-6- and IL-8-dependent mechanisms [20,21]. Under sufficient level, TNF-α has been also demonstrated to improve the efficacy of immunotherapy in tumors and initiate the apoptosis of tumor endothelial cells via the ligation of the TNFR1 [22,23]. Hence, achieving better understanding of the interplay between MSCs and immune systems would aid further development of cancer clinical therapy.

In this study, we hypothesized that TNF-α can activate the immunoregulatory potential of adipose-derived stem cells (ADSCs). Under sufficient TNF-α stimulation, we identified ADSCs’ stemness characteristics and immunomodulatory potential. Further, we cultured ADSCs with the T24 BC cell medium to determine whether ADSCs are involved in tumorigenesis, as a safety test for a future clinical trial for BC therapy.

## 2. Results and Discussion

### 2.1. Embryonic Stem Cell (ESC) Characteristics of ADSCs

**Figure 1:**
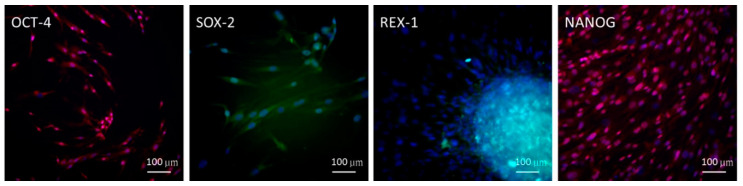
Embryonic stem cell characteristics of adipose-derived stem cells (ADSCs), shown by positive immunofluorescence staining results for OCT-4, SOX-2, REX-1, and NANOG expression (scale bar = 100 μm).

To detect the stemness characteristics of the ADSCs isolated, immunofluorescence staining with antibodies was performed for the ADSCs. The immunophenotype results were positive for embryonic stem cell (ESC) stemness-related markers, that is, octamer-binding transcription factor 4 (OCT-4), SRY-box transcription factor 2 (SOX-2), reduced expression protein 1 (REX-1), and NANOG homeobox (NANOG) (Figure 1). This result demonstrated that ADSCs used in this study fulfilled the definition of MSCs, representing the substantial circuitry of self-renewal.

### 2.2. Stimulation of ADSC Proliferation by TNF-α Treatment

**Figure 2:**
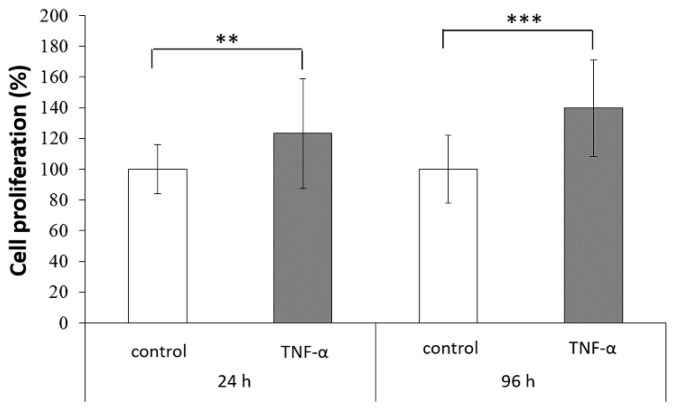
Proliferation of ADSCs treated with tumor necrosis factor (TNF)-α for 24 or 96 h (** *p* < 0.01, *** *p* < 0.001).

The effect of TNF-α treatment on ADSC proliferation was assessed using a 3-(4,5-dimethylthiazol-2-yl)-2,5-diphenyltetrazolium bromide (MTT) assay. Compared with the control group, ADSCs showed significantly higher proliferation after TNF-α treatment, particularly after 96 h of incubation (Figure 2). TNF-α, one of the main cytokines involved in acute inflammation, has receptors that induce multiple biological responses, including stimulation of cell growth, apoptosis, and differentiation [24]. This increased proliferation might be advantageous for tissue repair, which involves replacement of injured cells and secretion of trophic factors.

### 2.3. Activation of NF-κB Signaling in ADSCs for Cell Proliferation and Differentiation

**Figure 3:**
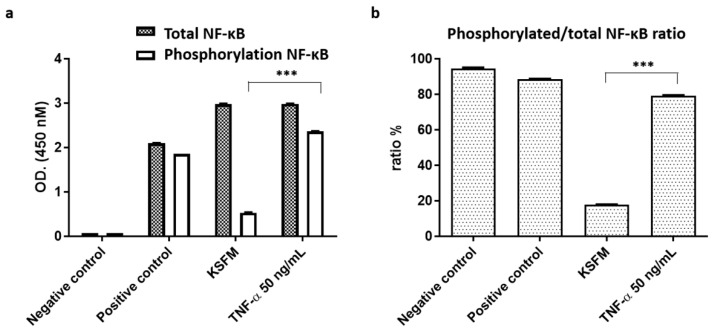
Nuclear factor (NF)-κB expression in ADSCs treated with tumor necrosis factor (TNF)-α. (**a**) Quantification of total and phosphorylation of NF-κB, respectively. (**b**) Quantification of phosphorylated/total NF-κB ratio (OD = optical density, KSFM = keratinocyte serum-free medium, *** *p* < 0.001).

In this study, 50 ng·mL^−1^ was used as the optimum TNF-α concentration for ADSC proliferation. The results demonstrated that ADSCs treated with this concentration could significantly activate the nuclear factor (NF)-κB signaling pathway (Figure 3a,b), which is involved in the subsequent differentiation-related experiments. NF-κB activation is vital for regulating multiple aspects of immune functions and inflammatory responses [25,26]. The TNF family activates the NF-κB pathway, which induces rapid transcription of genes that regulate inflammation, cell survival, proliferation, and differentiation. Newly synthesized IκBα thus enters the nucleus, binds to NF-κB, and translocates it to the cytoplasm, terminating NF-κB-directed transcription. Ubiquitination and proteasomal degradation of DNA bound NF-κB subunits have recently been identified as a major limiting factor of NF-κB mediated transcription independent of IκBα. It is important that their effects on NF-κB and DNA binding appear to be critical for tuning cytokine-induced NF-κB target gene expression and cell proliferation and differentiation, respectively [27]. Hence, we suggest that one of the reasons TNF-α can increase the immune response and cell growth of the ADSCs is through phosphorylating NF-κB signaling pathways in TNF-α stimulation rather than keratinocyte serum-free medium (KSFM) of cell culture medium.

### 2.4. Immunomodulatory Response of ADSCs Treated with TNF-α

**Figure 4:**
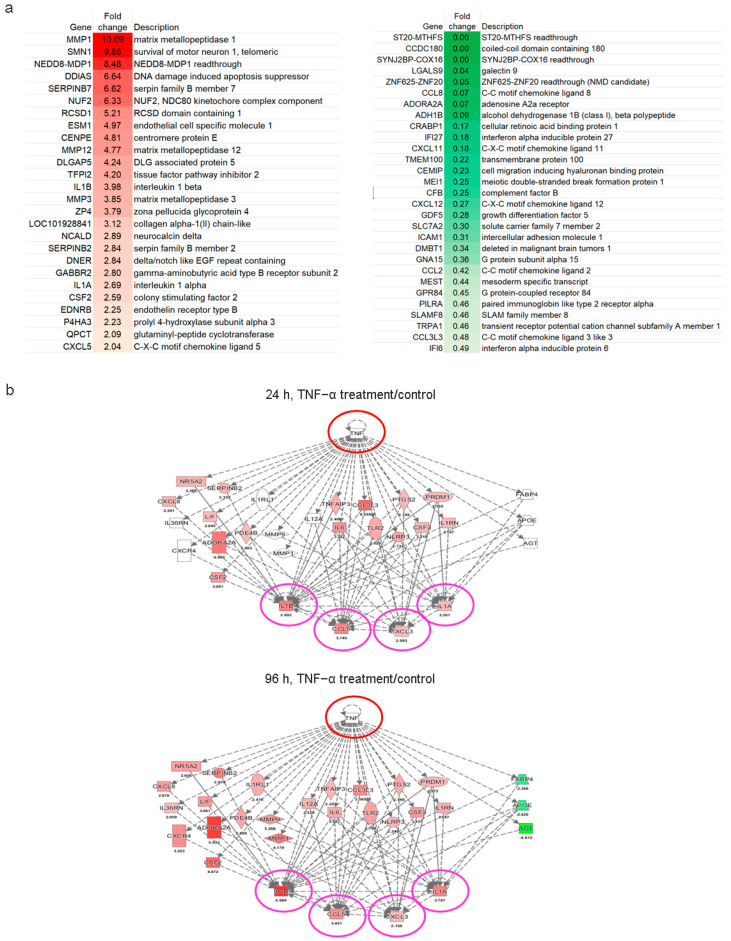
RNA sequencing–based gene expression profiling of ADSCs subjected to TNF-α treatment. (**a**) Regulated progenitor genes in TNF-α treated ADSCs for 24 h. (**b**) Pathway analysis of representative genes in ADSCs that responded to TNF-α treatment performed for 24 h and 96 h, proposed using the Ingenuity software (Qiagen, Hilden, Germany). Red and green colored shapes indicate up- and downregulated gene expression, respectively. The red and pink circles indicate the stimulator (TNF-α) and key mediators in the signaling network (IL-1A, IL-1B, CCL5, and CXCL3). The number under each symbol indicated on the maps is the ratio fold change between the treatment and control groups.

RNA sequencing was performed to examine the immunomodulatory response of ADSCs after TNF-α treatment; up- and downregulated progenitor genes are shown in Figure 4. Our data indicate that TNF-α treatment activated ADSCs during the inflammation response and elevated their mitosis-related cell proliferation property induction, such as G protein-coupled receptor signal transduction, regulation of extracellular matrix remodeling and calcium-dependent homeostasis of cells, precursor cell development, DNA-binding transcription factor activity, and obsolete signal transducer activity. RNAseq analysis showed that 25 genes, that is, MMP1, SMN1, NEDD8, SERPINB7, NUF2, RSCD1, ESM1, CENPE, MMP12, DLGAP5, TFPI2, IL1B, MMP3, ZP4, LOC101928841, NCALD, SERPINB2, DNER, GABBR2, IL1A, CSF2, EDNRB, P4HA3, QPCT, and CXCL5, of a total of 184 differentially expressed genes (DEGs) were significantly upregulated (*p* < 0.05 or *p* < 0.01; Figure 4a). Furthermore, 29 genes, that is, ST20-MTHFS, CCDC180, SYNJ2BP-COX16, LGALS9, ZNF625-ZNF20, CCL8, ADORA2A, ADH1B, CRABP1, IFI27, CXCL11, TMEM100, CEMIP, MEI1, CFB, CXCL12, GDF5, SLC7A2, ICAM1, DMBT1, GNA15, CCL2, MEST, GPR84, PILRA, SLAMF8, TRPA1, CCL3L3, and IFI6 were markedly downregulated. Transcriptomic analysis showed that TNF-α treatment increased IL-1A, IL-1B, CCL5, and CXCL3 expression levels after 96 h (Figure 4b). These results showed that TNF-α may expand its pro-inflammatory effect on ADSCs via the secretion of various cytokines or chemokines. Increasing studies have reported that MSCs can regulate immune function [11,13]. In the current study, we found that ADSCs significantly upregulated genes associated with leukocyte differentiation in the inflammatory microenvironment, indicating that ADSCs could release soluble factors via paracrine on immune cells to promote leukocyte proliferation and differentiation [28].

**Figure 5:**
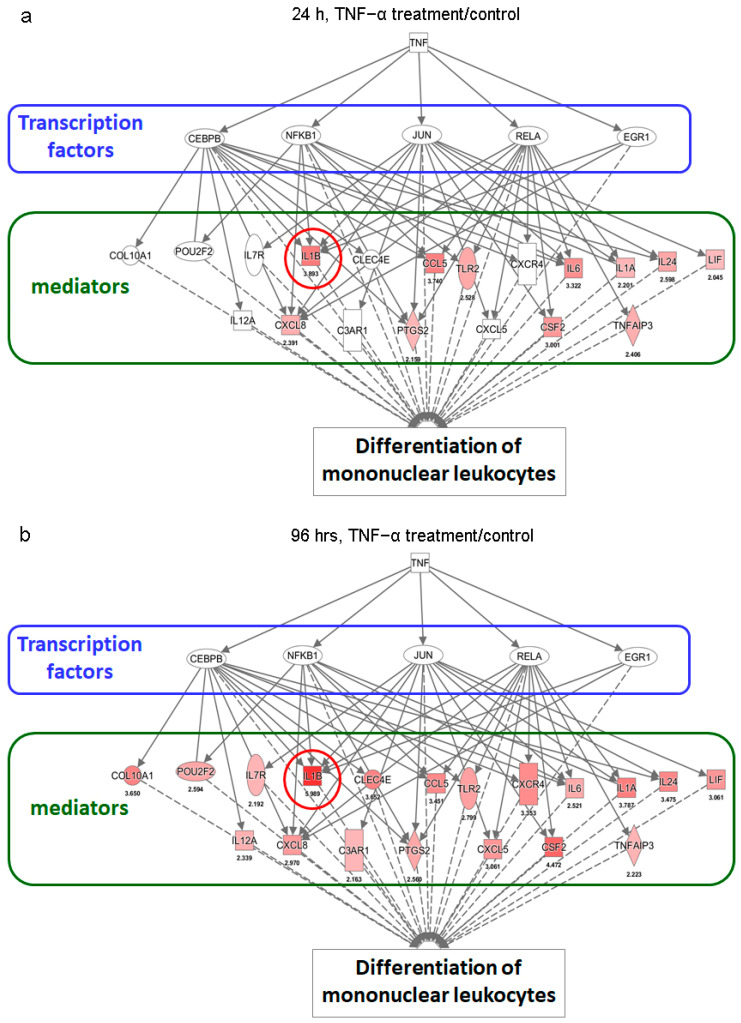
Leukocyte differentiation-related pathways in ADSCs involving putative signaling networks of CEBPB-, NFKB1-, JUN-, RELA-, and EGR1-mediated transcriptional activities in response to TNF-α stimulation for 24 (**a**) and 96 h (**b**), proposed using the Ingenuity software (Qiagen, Hilden, Germany). The red circles indicate changes in IL-1B expression levels in the TNF-α-induced signaling network. The number under each symbol indicated on the maps is the ratio fold change between the treatment and control groups.

Furthermore, analysis of the gene expression profile of ADSCs after TNF-α treatment showed increased expression levels of many leukocyte differentiation–related genes, especially IL-1B (Figure 5a,b). This effect was more obvious after 96 h of TNF-α treatment than 24 h. The findings also indicated that some transcription factors, including CEBPB, NFKB1, JUN, RELA, and EGR1, mediated the TNF-α-induced differentiation of ADSCs into mononuclear leukocytes. These included regulons centered on NFKB1, IRF8, RELA, RELB, IRF7, and other transcription factors that are strongly associated with monocytic lineage differentiation and whose interaction may be relevant to myeloid cell survival and development [29,30]. Thus, these results suggest that TNF-α treatment would enhance the potential of ADSCs to differentiate into immune cells.

### 2.5. Immunomodulatory and Antitumor Potentials of ADSCs Treated with TNF-α

**Figure 6:**
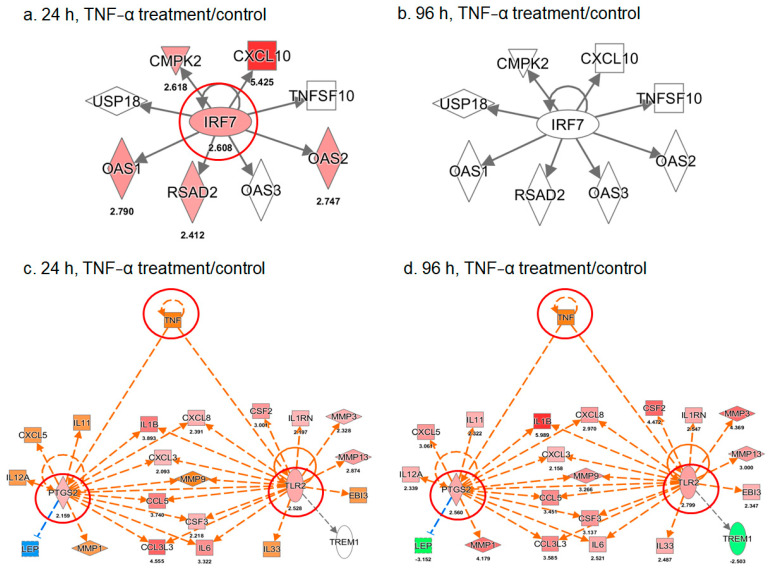
Effect of TNF-α treatment on IRF7-regulated gene expression (**a**,**b**) and TLR2- or PTGS2-mediated pathways (**c**,**d**) in ADSCs. A putative signaling network for the expression of genes that responded to TNF-α stimulation of ADSCs for 24 and 96 h were proposed using the Ingenuity software. The red and green symbols indicate up- and downregulated gene expression, respectively, while the orange and blue symbols represent nonresponsive genes; however, they were predicted as activated and inhibited molecules in these networks. The number under each symbol indicated on the maps is the ratio fold change between the treatment and control groups.

TNF-α treatment for 24 h increased IRF7 expression and consistently upregulated the expression of its downstream genes, such as CXCL10 (Figure 6a). However, this effect was not detected in ADSCs treated with TNF-α for 96 h (Figure 6b). These results suggest that TNF-α treatment transiently increased the signaling activity of type I interferons in ADSCs at an early stage, indicating immunomodulatory and antitumor characteristics. Further analysis showed the stimulatory effects of TNF-α treatment on TLR2 and PTGS2 expression in ADSCs (Figure 6c,d). Consistent with this finding, the expression profiles of their downstream genes also supported the activation of TLR2 and PTGS2; the effect was more obvious after 96 h of TNF-α treatment than 24 h. In line with the activation of type I interferons, these results indicate that TNF-α may have a pro-inflammatory effect on ADSCs via the TLR2- or PTGS2-mediated pathways and may promote antitumor immunity. TLRs have been identified on several stem or progenitor cells with substantial roles related to basal motility, self-renewal, differentiation potential, and immunomodulation [31]. In addition to the ability of TLRs to recognize pathogen-associated molecular patterns, they also recognize endogenous ligands such as alarmins, which are associated with autoimmune diseases and cancer [32,33]. Among the TLRs, TLR2 is strongly involved in the reparative properties of stem/progenitor cells because it secretes several reparative cytokines and chemokines, including IL-6, IL-8, C3, MCP-1, inhibin-A, and decorin, and is responsible for activation of the NF-κB signaling pathway [34]. In addition, PTGS2 signaling in tumor cells regulates and sensitizes tumors to immune therapy, representing a possible therapeutic option for immunotherapy-refractory cancers [35].

### 2.6. ADSC-Mediated Immunosuppression as a Modulator of Immune Responses

**Figure 7:**
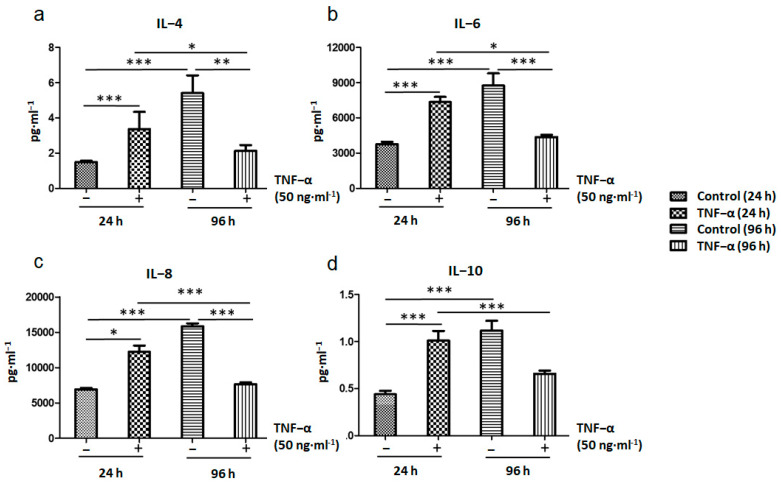
Inflammatory cytokine expression including (**a**) IL-4, (**b**) IL-6, (**c**) IL-8, and (**d**) IL-10 in TNF-α-treated ADSCs after 24 and 96 h (* *p* < 0.05, ** *p* < 0.01, *** *p* < 0.001).

During the first 24 h of TNF-α treatment, the expression of pro-inflammatory cytokines such as IL-4, IL-6, and IL-8 increased in ADSCs; however, it decreased after 96 h of treatment (Figure 7a–c). The same trend was observed for the expression of the anti-inflammatory factor IL-10 (Figure 7d). MSCs play a key role in the inflammatory microenvironment; they trigger completely opposite responses in cells in response to different types of inflammation. It was suggested that MSCs release several cytokines such as IL-2, IL-6, IL-8, SDF-1, and TNF to promote the migration and differentiation of tissue cells [36,37]. IL-8 concentration is relatively low under general physiological conditions; however, IL-8 can be very rapidly induced by pro-inflammatory cytokines such as TNF-α and IL-1B [38]. MSCs also alter T helper cell functions, for example, they increase IL-4 secretion [13] in Th2 cells and decrease IFNγ, IL-2, and TNF-α production by Th1 cells. MSCs delay neutrophil apoptosis via an IL-6–mediated mechanism that linked to reactive oxygen species reduction [39]. MSCs highly inhibit natural killer (NK) cell proliferation; this inhibitory effect is related to MSC-secreted prostaglandin E2 (PGE2), IDO, TGF-β1, IL-6, and nitric oxide expression [40,41]. Nemeth et al. recently reported that lipopolysaccharide and TNF-α induce high secretion of PGE2 by MSCs during septic situation; furthermore, they can reprogram monocytes and macrophages for secretion of large amounts of IL-10 [42]. The IL-10 released seems to prevent neutrophil migration into tissue and oxidative damage, thereby mitigating multi-organ damage. Hence, the results indicate that MSCs may modulate the innate immune response and improve survival by preventing and reversing sepsis. We have later discussed our findings, suggesting the mechanisms underlying the immunomodulatory capacities of MSCs and their applications in relation to the subsequent experiments on BC.

**Figure 8:**
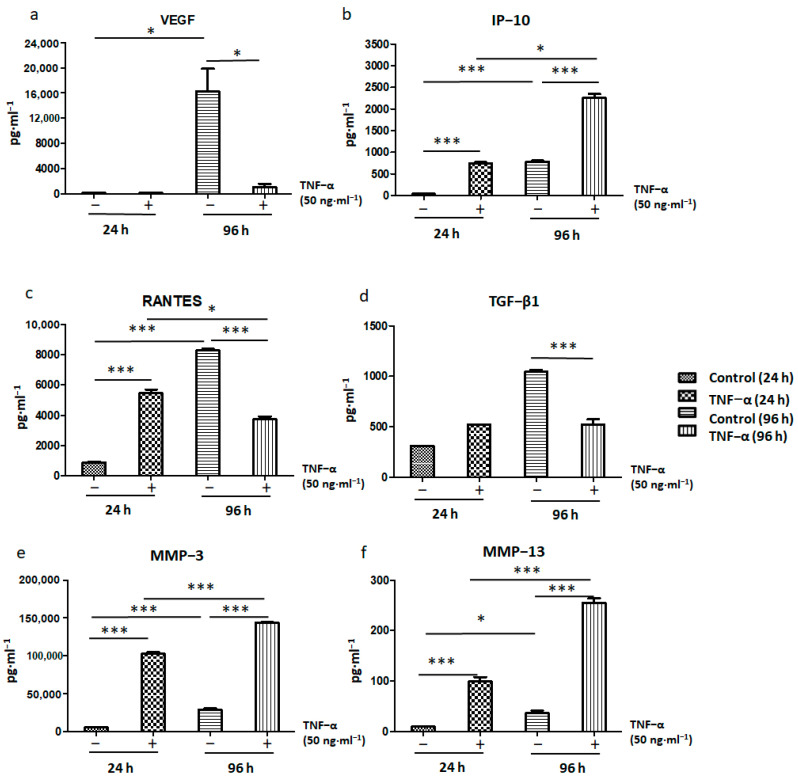
Immune-mediated responses to tumorigenesis inhibition of (**a**) VEGF, (**b**) IP-10, (**c**) RANTES, (**d**) TGF-β1, (**e**) MMP-3, and (**f**) MMP-13 by TNF-α-treated ADSCs after 24 and 96 h (* *p* < 0.05, *** *p* < 0.001).

Concerns still exist regarding the possible contribution of living MSCs to tumorigenesis, thereby indicating potential risks of MSC treatment in terms of tumor induction. Therefore, we investigated whether ADSCs can inhibit tumor growth after TNF-α treatment. The results showed that the expression of pro-inflammatory cytokines related to tumor growth, such as VEGF, RANTES, and TGF-β1, decreased after treatment of ADSCs with TNF-α for 96 h (Figure 8a,c,d). However, expression of the tumor suppressor chemokine IP-10 (Figure 8b) significantly increased. In addition, the expression of matrix metallopeptidases (MMPs) such as MMP3 and MMP13 (Figure 8e,f), which promote stem cell migration and homing ability, also increased under TNF-α treatment.

MSCs directly or indirectly exert anti-inflammatory and immunosuppressive effects by releasing various chemokines and inflammatory cytokines. Cytokine-dependent pathways indicate an essential part of T cells and monocytes regulatory. The current findings showed that MSC-derived VEGF may mediate the differentiation of endothelial progenitor cells into endothelial cells. To explore the regulatory roles of paracrine pathways in this process, IP-10/CXCL-10 are considered as chemotactic factors for human MSCs. Human MSCs migrate to demyelinated lesions in response to chemokines such as SDF-1, MCP-1, RANTES, MIP-1α, and IP-10 [43]. IP-10 is an antitumor agent that promotes damage in established tumor vasculature, which is attributed to its biological functions, such as the following: inhibition of colony formation by human hematopoietic cells; chemoattraction of human monocytes, activated T cells, and NK cells; stimulation of T cell adhesion to endothelial cells and of NK cell-mediated cytolysis; and inhibition of tumor angiogenesis [44,45,46,47]. However, IP-10 induces antitumor and antimetastatic activities via different immunological and antiangiogenic mechanisms.

Increasing evidence has shown the pro-tumorigenic roles of MMPs in cancer; however, the MMPs involved in cancer are inducible and also participate in the normal tissue homeostasis and remodeling [48]. In squamous cell carcinoma, different MMP family members have distinctly shown both pro- and antitumor activities [49]. Therefore, inhibition of MMPs with established pro-tumorigenic functions would need to be selective; these MMPs could also be used as antitumor agents during the correct stage of progression [50]. The current study has highlighted the antitumor properties of MMP3 in relation to tumorigenic keratinocyte differentiation and tumor reduction. We found that ADSCs failed to reduce MMP expression (Figure 8e,f). MMPs comprise a family of extracellular matrix-degrading proteinases implicated in various normal and pathological cellular processes, including embryogenesis, angiogenesis, wound healing, and cancer [51]. However, stem cells exhibit homing to their niches, and adequate MMPs are important for this mobilization and homing. MMPs are proteolytic mediators of extracellular matrix remodeling and may thereby enable the microenvironment alterations required to trigger cellular differentiation during stem cell development [52]. MMPs cause extracellular matrix cleavage and rearrangement and are associated with remodeling of stem cell niches.

Further, our experiments showed that TGF-β1 expression increased on treatment with TNF-α for 24 h but decreased at 96 h (Figure 8d). These data showed that TGF-β1–induced cell proliferation and angiogenesis associated with antitumor effects decreased when ADSCs were treated with TNF-α for longer periods. Recent research has shown that the VEGF receptor is regulated by TGF-β1 via parallel but distinct Smad pathways [53]. The VEGF expression level significantly differed between 24 and 96 h (Figure 8a). Thus, the findings suggested that the activation of ADSCs by TNF-α in tumor suppression experiments depended on the duration of activation. Signaling by TGF-β family ligands plays key roles in cell differentiation and proliferation and is important for many stem cell types. Among the TGF-β family proteins, signaling by TGF-β and activin proteins is essential for maintaining the pluripotency of human ESCs [54] and mouse epiblast-derived stem cells (EpiSCs) and helps define the differentiation potential and proliferation of these cell types. Results similar to those for TGF-β1 were obtained for pro-inflammatory factors such as RANTES (Figure 8c). Inhibition of proinflammatory RANTES expression by TGF-β1 has been reported to be mediated by β-catenin-triggered blockade of NF-κB signaling [55]. MSCs exist in virtually all tissues of the body and express multiple receptor types that sensitively detect tissue homeostasis. Stimulation of different TLRs in MSCs causes their polarization toward an anti-inflammatory phenotype characterized by increased production of the immunoregulatory factors RANTES and IP-10 [56]. On the basis of the aforementioned results, we hypothesize that if ADSCs are activated by TNF-α, they would have the potential to inhibit tumor growth during the immune response.

### 2.7. Cell Ability of TNF-α Treated ADSCs Cultured with Bladder Cancer Cells (T24 Cells) Medium

**Figure 9:**
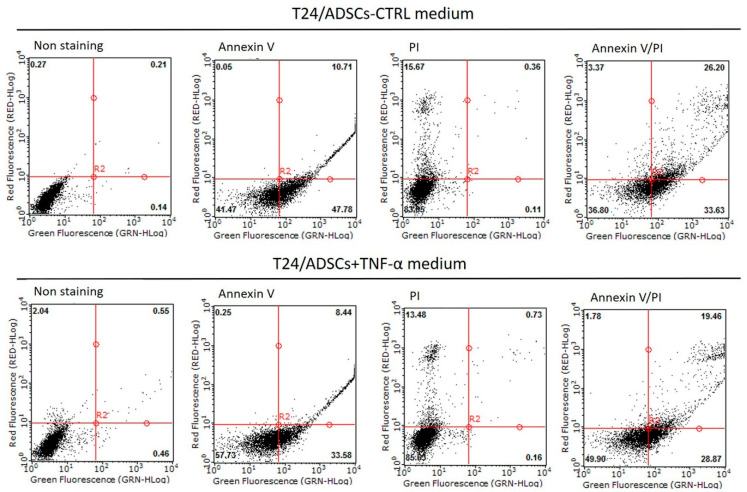
Flow cytometry analysis of ADSCs and TNF-α-treated ADSCs cultured with bladder cancer cells (T24 cells) medium for 24 h.

**Figure 10:**
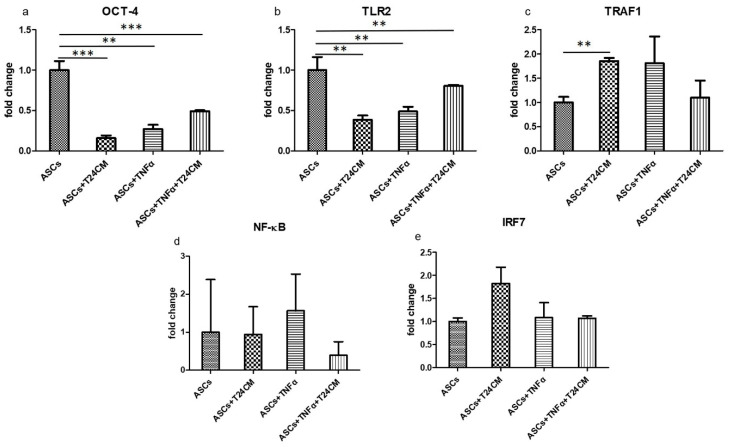
Activation of genes downstream of the Toll-like receptor signaling pathway including (**a**) OCT-4, (**b**) TLR2, (**c**) TRAF1, (**d**) NF-κB, and (**e**) IRF7 during the incubation of ADSCs and TNF-α-treated ADSCs with bladder cancer cells (T24 cells) medium for 24 h. (** *p* < 0.01, *** *p* < 0.001).

Flow cytometric analysis for the viable, apoptotic, and necrotic cell populations of ADSCs and TNF-α-treated ADSCs cultured with T24 cells medium showed that 26.20% and 19.46%, respectively, of these cell populations exhibited necrosis (Figure 9). Furthermore, the expression of the self-renewing marker OCT-4 of ADSCs decreased under both conditions—ADSC cultured with T24 cells medium and TNF-α treatment; TLR2 expression showed a similar trend (Figure 10a,b). Accumulating evidence related to the effects of TLR agonists on the functions of MSCs, such as hematopoietic stem cells, suggests that TLR signals influence MSC proliferation and differentiation, leading to rapid modulation of the immune response [57]. Furthermore, our results showed that the expression of the genes downstream of the TLR signaling pathway, such as TRAF1, NF-kB, and IRF7, was upregulated, in both cases, that is, whether ADSCs were cultured with T24 cells medium or were treated with TNF-α (Figure 10c–e). We found that TLR signaling of ADSCs was significantly less quiescent on incubation with T24 cells medium. In contrast, the expression of the genes downstream of the TLR signaling pathway increased in ADSCs upon TNF-α exposure. These data indicated that ADSCs had distinct immunomodulatory effects depending on the regulatory factors related to TLR signaling during tumorigenesis with the bladder cell microenvironment.

**Figure 11:**
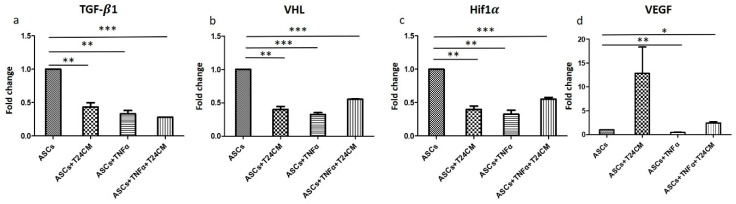
Downregulation of tumorigenesis-related growth factors including (**a**) TGF-β1, (**b**) VHL, (**c**) Hif1α, and (**d**) VEGF in ADSCs and TNF-α-treated ADSCs cultured with bladder cancer cells (T24 cells) medium for 24 h (* *p* < 0.05, ** *p* < 0.01, *** *p* < 0.001).

Moreover, TGF-β1, VHL, Hif1α, and VEGF expression (Figure 11a–d) decreased on cultured of ADSCs with T24 cells medium and on incubation with TNF-α. The findings suggest that ADSCs generated effective ability to inhibit tumorigenesis. Considerable research has shown that ESCs have antitumor effects, that is, ESCs may generate soluble factors that arrest or slow the population growth of tumors [58]. In addition, stem cells have the potential to reprogram cancer cells into a less invasive phenotype and may even prevent tumorigenesis and metastasis [59,60].

## 3. Materials and Methods

### 3.1. ADSC Isolation, Culture, and Stimulation with TNF-α and Bladder Cancer Cells (T24 Cells) Medium

Adipose tissue was collected by clinical surgery, as approved by the Ethics Committee of the Institutional Review Board of Tri-Service General Hospital (Taipei, Taiwan, R.O.C.; IRB approval number: 1-103-05-157). After the samples (~1–3 mL) were isolated, they were incubated immediately with transfer buffer (0.1 M phosphate-buffered saline [PBS], 1% penicillin/streptomycin, and 0.1% glucose). The tissue was then cut into 1-mm diameter sections, transferred into 10 mL Dulbecco’s modified Eagle’s medium (DMEM) containing 0.1% collagenase, and incubated for 1 day in a 37 °C incubator. The tissue sections were then transferred to a DMEM/10% fetal bovine serum (FBS; Gibco, Thermo Fisher Scientific, Waltham, MA, USA) solution for another 1 day in a 37 ℃ incubator, following which the cells were collected by centrifugation at 500× *g* for 5 min. The resulting pellet was suspended in a keratinocyte serum-free medium (KSFM, Gibco, Thermo Fisher Scientific) containing 5% FBS and the antioxidants *N*-acetylcysteine and l-ascorbic acid-2-phosphate in a 25 cm^2^ flask containing medium and incubated under 5% CO_2_ at 37 ℃. After 2–4 days of incubation (depending on the cell growth rate), the primary cells were collected after changing the medium. In this study, we used ADSCs from passage 75 (internal number: LSCTR-98-03-O-p75). For TNF-α stimulation, the ADSCs were seeded in 96-well plates; after 24 h, the cells were treated with 50 ng·mL^−1^ TNF-α. This cell stimulation with TNF-α lasted for 24 or 96 h. Bladder cancer cells (T24 cell line) were obtained from ATCC, Rockville, MA, USA. RPMI culture medium contained 10% FBS was used for the maintenance of T24 cells. For the interaction with T24 cells medium, both ADSCs and TNF-α-treated ADSCs were incubated with T24 cells culture medium for 24 h. This cell stimulation was used for flow cytometry and real-time PCR experiments.

### 3.2. Cell Proliferation

The proliferation of TNF-α-treated ADSCs was analyzed with the MTT colorimetric assay, using TNF-α treatment for 24 or 96 h. Cells were cultured under 5% CO_2_ at 37 °C in KSFM supplemented with 5% FBS. Cells incubated without TNF-α served as the control. The culture medium was replaced every 2 days during incubation. After each time point, the medium from each group was removed and replaced with the MTT solution (5 mg·mL^−1^) and incubated at 37 ℃ for 4 h. Then, the supernatant was carefully removed, and the DMSO solution was added to dissolve the crystals by gentle agitation for 10 min at room temperature. The absorbance for each group at 570 nm was read on a microplate reader (Bio-Tek ELX-800; BioTek, Winooski, VT, USA). The tests were performed in triplicate.

### 3.3. Immunofluorescence Staining

Non-treated ADSCs were grown in a slide flask (Nalge Nunc International, Rochester, NY, USA) for 24 or 96 h. The cells were then fixed with 4% paraformaldehyde, washed with PBS, and incubated with primary antibodies (OCT-4 (monoclonal mouse, sc-5279), SOX-2 (monoclonal mouse, sc-365823), REX-1 (monoclonal mouse, sc-377095), and NANOG (monoclonal mouse, sc-376915); 1:500 dilution; all from Santa Cruz Laboratories, Dallas, TX, USA) for 2 h at 37 °C. Subsequently, the samples were washed with PBS, incubated with fluorescein isothiocyanate-labeled secondary antibodies anti-rabbit and Texas Red–conjugated phalloidin anti-mouse (1:1000 dilution; all from Sigma-Aldrich, St. Louis, MO, USA) for 2 h at 37 °C, and subjected to Hoechst 33342 staining (1:5000 dilution; AnaSpec, Fremont, CA, USA) for 15 min for visualizing the nuclei. Fluorescence microscopic images were captured using a fluorescent microscope (Axio Lab.A1, Carl Zeiss AG, Oberkochen, Germany) with the Zeiss AxioCam ICm1 camera (Carl Zeiss AG).

### 3.4. Enzyme-Linked Immunosorbent Assay (ELISA)

ELISA was performed for NF-κB p65 ELISA Kit (Cat. No. ab176663, Abcam, Burlingame, CA, USA), following the manufacturer’s protocol. Briefly, 500 μL of cell culture supernatants were centrifuged at 2000× *g* for 5 min to remove particulates before being collected for further processing. Measurement of soluble factors in cell culture media was completed on collected supernatants from three independent experiments. The optical density was measured at 450 nm using a BioTek PowerWave 340 microplate spectrophotometer (BioTek Instruments Inc., Winooski, VT, USA). Concentrations of the cytokine in the samples were determined using a standard curve of known concentrations from the standard sample provided by the kit. For each independent experiment, at least two technical replicates were combined for analysis.

### 3.5. Flow Cytometry

ADSCs were collected and fixed with alcohol–acetic acid solution (95% alcohol + 5% acetic acid) for 5 min. After the ADSCs were rinsed with PBS and 0.05% NP-40 solution (diluted with PBS), they were blocked with 2% FBS for 10 min. Subsequently, they were treated using the Annexin V/propidium iodide (PI) double staining kit (eBiosciences, San Diego, CA, USA) following the manufacturer’s instructions, and flow cytometry was performed (BD FACSCalibur, BD Biosciences, Franklin Lakes, NJ, USA). PI is widely used in conjunction with Annexin V to determine if cells are viable, apoptotic, or necrotic, on the basis of differences in plasma membrane integrity and permeability. The flow cytometry data were plotted in two-dimensional dot plots for PI versus Annexin V-fluorescein isothiocyante (FITC).

### 3.6. RNA Isolation, Real Time-PCR, and Library Construction for mRNA Sequencing

Total RNA was isolated using TRIzol reagent (Invitrogen, Carlsbad, CA, USA) and subsequently column-purified with a RNeasy mini kit (Qiagen, Hilden, Germany) as per the manufacturers’ instructions. The purified RNA was treated with DNase I (New England Biolabs, Ipswich, MA, USA) to remove the genomic DNA. RNA concentration and integrity of each sample were measured using an Agilent 2100 Bioanalyzer (Santa Clara, CA, USA). Expression analyses of the differentiation- and stemness-related inflammatory genes were performed using the SYBR Green PCR master mix (Roche, Basel, Switzerland) in a LightCycler 480 real-time PCR system (Roche) as per the manufacturer’s instructions. The results were normalized with respect to glyceraldehyde 3-phosphate dehydrogenase (GAPDH), according to the 2^−ΔΔC^_t_ method. For RNA sequencing, the cDNA libraries were prepared with 1 μg of starting total RNA using the Illumina TruSeq RNA library kit (Illumina Inc., San Diego, CA, USA), following the manufacturer’s instructions. The libraries were amplified via PCR (15 cycles), and the amplified library was sequenced using an Illumina HiSeq 2500 system together with Ingenuity software (Qiagen IPA) for data interpretation.

### 3.7. Luminex Cytokine Assay

The cytokine assay for IL-4, IL-6, IL-8, IL-10, IP-10, MMP-3, MMP-13, RANTES, VEGF, and TGFβ1 was used the Milliplex kit (EMD Millipore Corporation, Billerica, MA, USA) in 96-well plates according to the manufacturer’s instructions. All standards and samples with dyed antibody bound beads were used in duplicate, incubated overnight with shaking at 4 °C, and then treated with a biotinylated detection antibody cocktail for 1 h. After washing, the beads were incubated with a streptavidin-phycoerythrin complex, and the mean fluorescent intensities were quantified on a Luminex 200 analyzer (Luminex Corporation, Austin, TX, USA). The results were analyzed using the Bio-Plex Manager 6.0 software (Bio-Rad, Hercules, CA, USA).

### 3.8. Statistical Analysis

Statistical analysis was performed using the Statistical Package for Social Science version 18 (SPSS, Chicago, IL, USA). The data were considered statistically significant when *p* was <0.05. One-way ANOVA was performed to assess significant differences in all quantitative data.

## 4. Conclusions

We conclude that the increased immunomodulatory effects of ADSCs induced by TNF-α treatment may be due to the inhibition of tumorigenesis-related factors and the immune responses. Our findings suggest that TNF-α can boost ADSCs with higher regenerative capacity and/or long-term survival, which would have broader applicability for BC therapeutic strategies in the clinical setting. Our study also provides insights that may help the development of a new intervention related to the antitumor application of ADSCs.

## Data Availability

Data are available upon request.

## Abbreviations

MSCs	Mesenchymal stem cells
TNF	Tumor necrosis factor
ADSCs	Adipose-derived stem cells
IFN	Interferon
IL	Interleukin
TLRs	Toll-like receptors
NF	Nuclear factor
BC	Bladder cancer
ESC	Embryonic stem cell
MTT	3-(4,5-dimethylthiazol-2-yl)-2,5-diphenyltetrazolium bromide
DEGs	Differentially expressed genes
PTGS2	Prostaglandin-Endoperoxide Synthase 2
IP-10	Interferon gamma-induced protein 10
NK	Natural killer
PGE2	Prostaglandin E2
MMPs	Matrix metallopeptidases
TGF-β1	Transforming growth factor beta 1
VEGF	Vascular endothelial growth factor
EpiSCs	Epiblast-derived stem cells
DMEM	Dulbecco’s modified Eagle’s medium
PBS	Phosphate-buffered saline
FBS	Fetal bovine serum
KSFM	Keratinocyte serum-free medium
PCR	Polymerase chain reaction
GAPDH	Glyceraldehyde 3-phosphate dehydrogenase
